# Oral-malodor measurement and intention to quit smoking in men: A before–after study

**DOI:** 10.18332/tid/168365

**Published:** 2023-07-19

**Authors:** Naoko Yatabe, Takashi Hanioka, Nao Suzuki, Atsushi Shimazu, Marie Naito

**Affiliations:** 1 Department of Preventive and Public Health Dentistry, Fukuoka Dental College, Fukuoka, Japan; 2 Department of Oral Health Sciences, Faculty of Health Care Sciences, Takarazuka University of Medical and Health Care, Takarazuka, Japan; 3 Oral Medicine Research Center, Fukuoka Dental College, Fukuoka, Japan

**Keywords:** motivation, oral malodor, smoking cessation, respiratory function, heated tobacco products

## Abstract

**INTRODUCTION:**

Few studies have examined the effect of feedback based on oral-malodor measurements on the motivation to quit smoking. Therefore, this study examined whether oral-malodor measurements were associated with the intention to quit smoking.

**METHODS:**

This retrospective, uncontrolled before–after study invited smokers to a workplace health event in 2019 and 2020 to motivate them to quit smoking. They attended seminars on oral health and smoking cessation aids, and then underwent respiratory function and oral-malodor measurements using exhaled and oral cavity air, respectively. Intention to quit smoking was evaluated by answers to questions regarding the intention to quit in the next 1 or 6 months in questionnaires collected before and after the event. This study analyzed 241 men aged 20–54 years (mean: 33.2 ± 10.5) to examine factors associated with the intention to quit in multivariable logistic regression analyses for age, tobacco type (cigarettes and heated-tobacco products), and category of tobacco consumption.

**RESULTS:**

Before the event, 8.7%, 17.0%, and 74.3% of smokers had intended to quit in the next month, the next six months, or had no intention to quit, respectively. After the event, the respective percentages were 17.8%, 26.6%, and 55.6%. A higher methyl mercaptan concentration, a volatile sulfide component of oral malodor, was significantly associated with the intention to quit in the next month (adjusted odds ratio, AOR=4.24; 95% CI: 1.52–11.8, p=0.006). The participants with higher daily tobacco consumption were less likely to acquire the intention to quit in the next six months (AOR=0.37; 95% CI: 0.15–0.92, p=0.032). Other variables, such as lung age deficit, exhaled CO concentration, and hydrogen sulfide concentration (another component of oral malodor), were not significantly associated.

**CONCLUSIONS:**

Oral-malodor measurement feedback may help motivate men to quit smoking in the next 1 month rather than in the next six months.

## INTRODUCTION

Causal associations of tobacco use with oral cancer and periodontal disease have been documented in developed^1^ and developing^2^ countries. Tobacco use increases the risks of dental caries, periodontal diseases, dental implant failure, tooth loss, and oral malodor^3^. Exposure to secondhand smoke also increases the risk of periodontitis^4^, oral cancer^5^, and dental caries in children^6^. The increased risk of periodontal disease and dental caries may be strengthened by smoking-related oral dysbiosis^7^, and early tooth eruption may mediate the risk of pediatric dental caries^8^. Because any form of tobacco use and any amount of tobacco exposure affect oral health, brief tobacco interventions and oral health programs in primary care are recommended^9^.

The effects of interventions in dental settings on smoking cessation have been summarized through analyses of abstinence from long-term tobacco use with at least six months of follow-up to manage tobacco dependence^10^. In contrast to intensive interventions in dental settings, environmental strategies such as social pressure, tobacco taxes, and discomfort with tobacco users affect the intention to quit smoking^11^. Oral health professionals should use lower intensity tobacco interventions because of their relevance to the nature of dental treatment in primary care^12^.

The effects of interventions on the motivation to quit smoking in dental settings have not been extensively investigated, although smoking has various consequences for oral health that can affect the motivation to quit smoking^13^. Because of its effect on personal relationships, health professionals focus on oral malodor as a consequence of smoking to motivate smokers to quit smoking. Novel tobacco products such as e-cigarettes and heated-tobacco products (HTPs) are spreading rapidly worldwide. These products generate little or no carbon monoxide (CO); notably, CO cannot be detected in exhaled air from users of these products. Furthermore, these products may have fewer malodor components because of reduced combustion.

In 76% of patients at a bad-breath clinic, oral malodor originated in the oral cavity^14^. Volatile-sulfur-compound concentrations in oral cavity air are associated with oral malodor^15^. Smoking is associated with oral malodor because of its effects on the tongue-surface microbiome and saliva content^16,17^. Breath-focused interventions, with measurements of oral malodor and respiratory function, were effective in enhancing the motivation to quit smoking among cigarette and HTP users^18^. However, other than using respiratory-function measurements, few studies have examined the effect of feedback on motivation to quit smoking using oral-malodor measurements. Therefore, this study examined whether oral-malodor measurements influenced the intention to quit smoking.

## METHODS

### Study population and design

This retrospective, uncontrolled before–after study used the records of smokers who participated in a worksite health event^18^. Smokers who worked at a base of the Japan Grand Self-Defense Force were invited to a 1-day smoking cessation event. Smoking status was determined in advance at a worksite health checkup. Participation in the event was voluntary, and 266 smokers participated. Data for 25 smokers were not analyzed because of incomplete data (19 smokers) and a small sample group (6 female smokers). Ultimately, data for 241 male smokers [aged 20–54 years (mean: 33.2 ± 10.5), participation rate 44.1%] were analyzed. The participants were asked not to eat, drink, or smoke for at least 1 hour before the event. They entered several booths designed to: increase awareness of the importance of quitting smoking, including a 20-min video presentation regarding various mouth-specific effects of smoking; have a group introduction to smoking-cessation medication; and undergo measurements of CO and respiratory function in exhaled air and oral malodor, using air in the oral cavity. Participation in the group seminar was not mandatory.

### Questionnaires related to smoking behaviors

The participants were provided with questionnaires that inquired about tobacco-product use, such as cigarettes and HTPs, daily consumption, and the duration of smoking. The intention to quit smoking was assessed using three possible responses: ‘I want to quit smoking immediately or within 1 month’; ‘I want to quit smoking within six months, but I do not plan to quit smoking within 1 month’; and ‘I do not plan to quit smoking within six months’. The corresponding levels of intention to quit smoking were categorized as intention to quit in the next month, intention to quit in the next six months, and no intention to quit smoking, respectively.

### Respiratory function measurements

The exhaled CO concentration was measured using a handheld, portable breath CO monitor (piCO Advance Smokerlyzer, Bedfont Scientific, Maidstone, England, UK) in accordance with the manufacturer’s instructions. The forced expiratory volume in 1 s (FEV_1_) was measured using a simple spirometer (Lung Age Monitor, Vitalograph, Buckingham, England, UK) in accordance with the manufacturer’s instructions. Lung age was calculated using FEV_1_, in combination with height and age. The lung-age deficit was calculated as the spirometric-derived lung age minus chronological age.

### Oral-malodor measurements

To measure oral malodor, air that had been retained in the oral cavity for 30 s was collected using a 1 mL syringe. Volatile sulfur compounds were measured using a portable sulfide monitor (Oral Chroma, Nissha FIS, Osaka, Japan). Hydrogen sulfide, methyl mercaptan, and dimethyl sulfide concentrations were determined. Dimethyl sulfide is found at high concentrations in patients with systemic diseases^19^ and should not be present at high concentrations in generally healthy Self-Defense Forces personnel. Hydrogen sulfide and methyl mercaptan are mainly derived from dental plaque^20^ and periodontal disease^21^, respectively.

### Blinded feedback to participants

Medical staff provided feedback regarding respiratory function measurements immediately after measurements, while dentists provided feedback regarding oral malodor. The feedback was calibrated using a manual detailing the measurements. The examiners were blinded to the questionnaire that asked the participants about their intention to quit to minimize potential bias.

### Statistical analysis

Two analytical models were constructed according to the level of intention to quit. The case group in each model comprised participants who acquired the intention to quit within the next 1 or 6 months, and the control group comprised those who did not acquire the intention after participating in the event. Participant characteristics were compared according to the intention to quit using the chi-squared test and Mann–Whitney U test, as appropriate. Then, univariable and multivariable analyses assessed associations between the acquisition of the intention to quit and explanatory variables. Odds ratios and 95% confidence intervals (CIs) in each model for intention to quit in the next 1 or 6 months after participation, relative to the control group, were calculated by univariable (ORs) and multivariable logistic regression (adjusted odds ratios, AORs) analyses. The explanatory variables were age group, tobacco variables (tobacco use categories, daily tobacco consumption, duration of smoking, and Brinkman index), exhaled air variables (lung age deficit and CO concentration), and oral cavity air variables (hydrogen sulfide and methyl mercaptan concentrations). The Brinkman index, used to determine the total amount of tobacco smoking, was calculated by multiplying the number of cigarettes/day by the number of years of smoking history. The median values of explanatory variables, except for the Brinkman index, were used as cut-off values for logistic regression analyses of each variable. The cut-off value for the Brinkman index was set at 200 because a Brinkman index ≥200 is the threshold for receiving universal health insurance coverage for smoking cessation treatment in Japan. Interactions between explanatory variables in each group were also evaluated. All analyses were performed using SPSS ver. 27 (IBM Japan, Tokyo), with a significant threshold of p<0.05 by a two-tailed test.

## RESULTS

### Participant characteristics

Table 1 summarizes the participant characteristics. The 241 participants included 169 (70.1%) exclusive cigarette smokers, 39 (16.2%) exclusive HTP users, and 33 (13.7%) users of both. Greater intention to quit smoking was significantly associated with younger age, lower daily tobacco consumption, a lower Brinkman index, and a lower CO concentration in exhaled air. Associations with other variables, including tobacco use categories, were not statistically significant.

**Table 1:** Comparisons of smoker characteristics according to the level of the intention to quit before interventions. This retrospective uncontrolled before–after study invited smokers to a workplace health event in 2019 and 2020 to motivate them to quit smoking

*Characteristics*	*Total (N=241) Median (IQR)*	*Level of intention to quit smoking*			*p*
		*In the next month (N=21) Median (IQR)*	*In the next 6 months (N=41) Median (IQR)*	*No intention to quit (N=179) Median (IQR)*	
**Age** (years)	30.0 (24.0–43.0)	26.0 (21.5–40.0)	28.0 (22.0–37.5)	31.0 (25.0–44.0)	0.036^a^
**Tobacco use categories,** n (%)					0.917^b^
Exclusive cigarette smoking	169 (70.1)	16 (76.2)	29 (70.7)	124 (69.3)	
Exclusive HTP use	39 (16.2)	2 (9.5)	6 (14.6)	31 (17.3)	
Dual cigarette and HTP use	33 (13.7)	3 (14.3)	6 (14.6)	24 (13.4)	
**Tobacco consumption**					
Daily tobacco consumption	15.0 (10.0–20.0)	10.0 (10.0–15.0)	15.0 (10.0–15.0)	15.0 (10.0–20.0)	0.002^a^
Duration of smoking (years)	10.0 (4.0–22.0)	6.0 (2.5–20.0)	6.0 (2.5–15.5)	11.0 (5.0–23.0)	0.014^a^
Brinkman index	150.0 (45.0–320.0)	50.0 (25.0–242.5)	80.0 (25.0–262.5)	180.0 (60.0–375.0)	0.001^a^
**Measurement of exhaled air**					
Lung age deficit (years)	22.0 (9.0–39.0)	20.0 (3.5– 30.5)	18.0 (11.0–43.5)	24.0 (9.0–39.0)	0.519^a^
CO concentration (ppm)	16.0 (9.0–22.0)	14.0 (9.0–17.0)	14.0 (7.5–18.5)	16.0 (9.0–23.0)	0.047^a^
**Measurement of oral cavity air**					
Hydrogen sulfide (ppb)	821.0 (663.0–1179.0)	708.0 (659.5–1042.0)	815.0 (666.0–1250.5)	833.0 (653.0–1198.0)	0.642^a^
Methyl mercaptan (ppb)	57.0 (28.0–95.5)	32.0 (19.0–77.5)	60.0 (35.0–96.5)	58.0 (28.0–99.0)	0.167^a^

HTPs: heated tobacco products.

a Tested using the Kruskal–Wallis test.

b Tested using the chi-squared test. IQR: interquartile range.

### The trajectory of the intention to quit smoking

**Figure 1:**
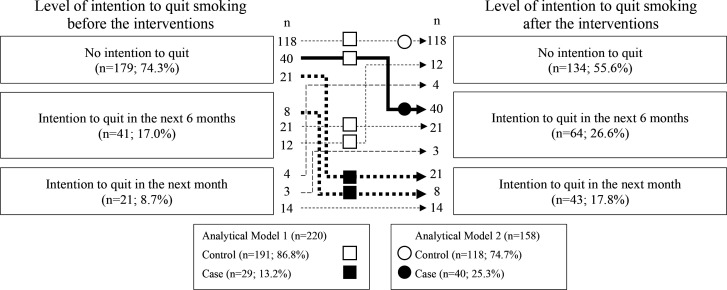
Numbers of smokers according to level of the intention to quit smoking before and after participating in a worksite health event, along with two analytical models to identify factors associated with the intention to quit smoking in the next 1 (Model 1) and 6 months (Model 2). The numbers of participants in each model were calculated according to the trajectory of the intention to quit smoking: Squares and circles represent the numbers of smokers analyzed in Models 1 and 2, respectively. Closed and open symbols indicate cases and controls, respectively, in each model. This retrospective uncontrolled before–after study invited smokers to a workplace health event in 2019 and 2020 to motivate them to quit smoking

Figure 1 shows the number of participants according to their intention to quit before and after participation. The numbers of participants in the case and control groups in each analytical model were calculated based on the numbers in Figure 1. Among the smokers, 21 (8.7%), 41 (17.0%), and 179 (74.3%) had the intention to quit in the next one month, intention to quit in the next six months, and no intention to quit smoking, respectively, before the health event; these numbers were 43 (17.8%), 64 (26.6%), and 134 (55.6%) after the event. Among smokers who had no intention to quit in the next one month before the event (n=220), 29 (13.2%) had the intention to quit in the next one month after the event; these participants formed the case group in analytical Model 1. Among smokers who had no intention to quit smoking in the six months before the event and smokers who had no intention to quit smoking in the one month after the event (n=158), 40 (25.3%) smokers acquired the intention to quit smoking in the next six months; formed the case group in analytical Model 2.

### Factors associated with the intention to quit smoking in the next month

Table 2 shows the ORs and 95% CIs of the variables in Model 1 for the acquisition of intention to quit smoking in the next month after participation in the event. In univariable analyses, lower daily tobacco consumption, a lower Brinkman index, a lower exhaled CO concentration, and a higher methyl mercaptan concentration in oral cavity air were significantly associated with the intention to quit smoking in the next month. In multivariable logistic regression analysis, the association was significant only for the methyl mercaptan concentration in oral cavity air. The adjusted odds ratio (AOR) of a higher methyl mercaptan concentration for intention to quit smoking in the next month relative to the control was 4.24 (95% CI: 1.52–11.84). Other variables were not significantly associated with the intention to quit smoking in the next month, including the use of HTPs and interactions between the variables. We conducted a sensitivity analysis by dividing the smokers into three groups according to the methyl mercaptan concentration. As the concentration increased, the proportions of smokers who acquired the intention to quit smoking in the next month increased by 8.3%, 11.0%, and 20.0%, respectively.

**Table 2:** Odds ratios for acquiring the intention to quit smoking in the next month after participating in the event. This retrospective uncontrolled before–after study invited smokers to a workplace health event in 2019 and 2020 to motivate them to quit smoking

*Variable*	*Acquisition rate % (n/N)*	*OR (95% CI)*	*p*	*AOR (95% CI)^a^*	*p*
**Age** (years)					
≥40	7.1 (5/70)	1		1	
<40	16.0 (24/150)	2.48 (0.90–6.79)	0.078	2.00 (0.40–10.31)	0.402
**Tobacco characteristics**					
**Tobacco use categories**					
Exclusive cigarette use	13.0 (20/153)	1		1	
HTP use	13.4 (9/67)	1.03 (0.44–2.40)	0.942	0.76 (0.31–1.90)	0.560
**Daily tobacco consumption** (pieces)					
≥15	6.2 (5/80)	1		1	
<15	17.1 (24/140)	3.10 (1.13–8.49)	0.027	2.42 (0.77–7.66)	0.131
**Duration of smoking** (years)					
≥10	8.5 (9/106)	1		1	
<10	17.5 (20/114)	2.29 (0.99–5.29)	0.052	1.66 (0.34–8.13)	0.534
**Brinkman index**					
≥200	7.6 (7/92)	1		1	
<200	17.2 (22/128)	2.52 (1.03–6.18)	0.043	0.72 (0.10–5.38)	0.751
**Characteristics of exhaled air**					
**Lung age deficit** (years)					
<24	13.0 (15/115)	1		1	
≥24	13.3 (14/105)	1.03 (0.47–2.24)	0.949	0.90 (0.39–2.09)	0.800
**Exhaled CO concentration** (ppm)					
≥16	7.0 (7/100)	1		1	
<16	18.3 (22/120)	2.98 (1.22–7.31)	0.017	2.24 (0.83–6.03)	0.112
**Characteristics of oral cavity air**					
**Hydrogen sulfide concentration** (ppb)					
<833	13.4 (15/112)	1		1	
≥833	13.0 (14/108)	0.96 (0.44–2.10)	0.925	0.54 (0.21–1.41)	0.206
**Methyl mercaptan concentration** (ppb)					
<57	7.5 (8/107)	1		1	
≥57	18.6 (21/113)	2.83 (1.19–6.69)	0.018	4.24 (1.52–11.84)	0.006

a AOR: adjusted odds ratio; adjusted for age group, tobacco variables (tobacco use categories, daily tobacco consumption, duration of smoking, and Brinkman index), exhaled air variables (lung age deficit and CO concentration), and oral cavity air variables (concentrations of hydrogen sulfide and methyl mercaptan). HTPs: heated tobacco products.

### Factors associated with the intention to quit smoking in the next six months

The ORs and 95% CIs of variables in Model 2 for the acquisition of intention to quit smoking in the next six months after participation in the event are shown in Table 3. In univariable analyses, no significant association was detected for any variable examined in this study. In multivariable logistic regression analysis, higher daily tobacco consumption (≥15 pieces) was significantly associated with the intention to quit smoking in the next 6 months after participation in the event. Other variables were not significantly associated with the intention to quit smoking in the next six months, including the use of HTPs and interactions between the variables.

**Table 3:** Odds ratios for acquiring the intention to quit smoking in the next 6 months after participating in the event. This retrospective uncontrolled before–after study invited smokers to a workplace health event in 2019 and 2020 to motivate them to quit smoking

*Characteristics*	*Acquisition rate % (n/N)*	*OR (95% CI)*	*p*	*AOR (95% CI)^a^*	*p*
**Age** (years)					
≥40	22.6 (12/53)	1		1	
<40	26.7 (28/105)	1.24 (0.57–2.70)	0.583	1.05 (0.29–3.78)	0.935
**Tobacco characteristics**					
**Tobacco use categories**					
Exclusive cigarette use	21.3 (23/108)	1		1	
HTP use	34.0 (17/50)	1.90 (0.90–4.01)	0.090	1.68 (0.75–3.77)	0.206
**Daily tobacco consumption** (pieces)					
≥15	32.1 (18/56)	1		1	
<15	21.6 (22/102)	0.58 (0.28–1.21)	0.146	0.37 (0.15–0.92)	0.032
**Duration of smoking** (years)					
≥10	23.3 (17/73)	1		1	
<10	27.1 (23/85)	1.22 (0.59–2.52)	0.052	0.72 (0.11–4.78)	0.730
**Brinkman index**					
≥200	22.7 (15/66)	1		1	
<200	27.2 (25/92)	1.27 (0.61–2.65)	0.527	2.38 (0.32–17.68)	0.396
**Characteristics of exhaled air**					
**Lung age deficit** (years)					
<24	25.0 (3/12)	1		1	
≥24	25.3 (37/146)	1.02 (0.26–3.96)	0.979	0.47 (0.21–1.04)	0.062
**Exhaled CO concentration** (ppm)					
≥16	19.4 (14/72)	1		1	
<16	30.2 (26/86)	1.80 (0.85–3.78)	0.123	2.04 (0.85–4.88)	0.109
**Characteristics of oral cavity air**					
**Hydrogen sulfide concentration** (ppb)					
<833	24.1 (20/83)	1		1	
≥833	26.7 (20/75)	1.15 (0.56–2.35)	0.711	0.74 (0.29–1.86)	0.519
**Methyl mercaptan concentration** (ppb)					
<57	21.4 (18/84)	1		1	
≥57	29.7 (22/74)	1.55 (0.75–3.19)	0.233	1.64 (0.64–4.17)	0.301

a AOR: adjusted odds ratio; adjusted for age, group, tobacco variables (tobacco use categories, daily tobacco consumption, duration of smoking, and Brinkman index), exhaled air variables (lung age deficit and CO concentration), and oral cavity air variables (concentrations of hydrogen sulfide and methyl mercaptan).HTPs: heated tobacco products.

## DISCUSSION

Smokers with higher methyl mercaptan concentrations, an indicator of oral malodor, were twice as likely to report an intention to quit smoking in the next month than smokers with lower methyl mercaptan concentrations. Among the variables measured, the methyl mercaptan concentration in oral cavity air was the only variable significantly associated with acquiring the intention to quit smoking. The results of the sensitivity analysis reinforce those of the multivariable analysis. Therefore, feedback on the methyl mercaptan concentration in oral cavity air may influence the intention to quit smoking. The hydrogen sulfide concentration might not have been significantly associated with quitting smoking because hydrogen sulfide concentrations may be decreased by tooth brushing rather than quitting smoking.

Oral malodor was measured using a point-of-care test, and smokers were informed of the results immediately after measurement. Bad breath generally affects interpersonal relationships negatively. Smokers with high methyl mercaptan concentrations received information about the potential for periodontal disease, which could lead to tooth loss^15^. Therefore, social and disease risk information may strongly motivate smoking cessation.

Oral malodor and xerostomia may be related to tobacco smoking. Xerostomia is common in smokers, including e-cigarette users^22^. The presence of xerostomia reduces the saliva-mediated self-cleaning action of the oral cavity^23^. When the self-cleaning action declines, various bacteria are likely to proliferate, thereby increasing bad breath^24^; the occurrence of bad breath leads to increases in the measured values. Smoke in cigarettes has the odor of aldehydes produced during combustion. Because these are not volatile compounds, they were not detected in this measurement. There are no reports of bad breath originating from HTPs. Comprehensive measurements of halitosis components are necessary to motivate more smokers to quit smoking.

Measurements of lung function via spirometry and assessments of lung age may help to motivate smoking cessation. In the present study, however, excess lung age and acquisition of the desire to quit smoking were not significantly associated with the acquisition of the intention to quit smoking. Smokers who smoke more may recognize that it is more challenging to quit smoking^25^; they also have worse spirometry and lung age results^26,27^. Therefore, long-term smokers with impaired respiratory function may have lower recognition of the important association between health behavior and illness. Alternatively, there may be a lack of information regarding potential confounders between two variables, such as nicotine dependence, which explains why knowledge of deterioration in lung function did not directly motivate smoking cessation. Oral measurement may be a novel means to motivate smokers to quit smoking.

The proportion of participants who had no intention to quit (74.3%) was similar to the previously reported proportion of Japanese smokers (73.9%) who did not plan to quit^28^. The proportions of smokers who had intention to quit in the next 1 and 6 months increased by approximately 2- and 1.6-fold, respectively, after participation in the event; the proportion of smokers who had no intention to quit decreased from 74.3% to 55.6%. Therefore, a breath-focused event may tentatively motivate smokers to quit smoking. The temporal increase in motivation to quit smoking should be followed up at the worksite dental clinic to increase long-term abstinence because repeated brief interventions during dental visits increased cessation attempts by 2.8-fold and prevented regression of the stage of behavior change by 2-fold^13^. To reduce smoking rates worldwide, such activities should continue, particularly in developing countries^29^.

This novel study examined factors associated with the intention to quit smoking among HTP users. However, there was no relationship between the type of smoking (i.e. HTPs vs cigarettes) and the intention to quit in the next month. Among HTP users, the intention to quit smoking may depend on two factors: 1) many HTP users believe that they are using HTPs to quit smoking; and 2) and they perceive that HTPs are less harmful than cigarettes^30^. HTP users are less willing to quit smoking altogether compared with cigarette users^31^. The most important reason smokers provided for switching from cigarettes to HTPs was that combustible cigarettes were bad for their health^32^. Therefore, HTP users may retain the intention to quit smoking altogether. Further studies are needed to clarify which trend is more prevalent among HTP users and which factors contribute to that trend.

Smokers who consumed less tobacco per day were more likely to acquire the intention to quit smoking in the next six months. Smokers who want to quit smoking in the next six months valued both quitting and continuing to smoke, whereas smokers who want to quit in the next one month had made up their minds and were looking for specific ways to quit smoking^33^. Smokers who consume less tobacco daily may not recognize the symptoms of altered respiratory function. Measurements of respiratory and oral functions and education at health events may facilitate the acquisition of the intention to quit smoking in the next six months.

### Limitations

All smokers analyzed in this study were men. The smoking rate in Japan is lower among women than men (8.1% vs 29.0% in 2018). Caution should be exercised when interpreting our results because of possible under-adjustment for unknown confounders and selection bias; our findings may not be generalizable across all occupational sectors or all smokers. Since the study was retrospective and the available data were limited, another significant factor may have been missed because of the small sample size.

## CONCLUSIONS

Interventions that used the health effects of dentistry and respiratory function assessments effectively motivated smoking cessation in men, including cessation among users of heated tobacco products. The effect was specific to smokers with higher concentrations of methyl mercaptan, an essential component of oral malodor, regardless of smoking type.

## Data Availability

The data supporting this research are available from the authors on reasonable request.
